# Re-Envisioning Tarascan Temporalities and Landscapes: Historical Being, Archaeological Representation, and Futurity in Past Social Processes

**DOI:** 10.1007/s10816-016-9279-x

**Published:** 2016-03-10

**Authors:** David L. Haskell, Christopher J. Stawski

**Affiliations:** 1 Department of Anthropology, The Ohio State University, 4034 Smith Laboratory, 174 W. 18th Ave., Columbus, OH 43210 USA; 2 Department of Anthropology, San Francisco State University, 1600 Holloway, SCI 377, San Francisco, CA 94132 USA

**Keywords:** Landscape, Phenomenology, GIS, State formation, Human-environment dynamics, Futurity

## Abstract

We apply a phenomenological perspective on landscape and geographic information system (GIS) applications in order to theorize how human perception and agency were likely implicated in processes of the formation of the late pre-Hispanic Tarascan State of West Central Mexico. The relatedness of landscape features in space or place-based perception has been well theorized; here, we further consider the relationality of places through time. In the changing landscape of the Lake Pátzcuaro Basin, the demographic and political core of the Tarascan State, temporality must have been vitally important to inhabitants of the basin. Utilizing GIS, we construct not only map-based analyses of the changing environment but also create viewsheds of past landscapes in order to see what past inhabitants of the basin would have seen in order to demonstrate that temporality would have been easily mapped in the landscape and its features. Finally, we discuss the role of temporality and cultural memory in an embodied landscape to model the various lake levels that past peoples could have anticipated through time based on their perceptions and memories.

**Electronic supplementary material:**

The online version of this article (doi:10.1007/s10816-016-9279-x) contains supplementary material, which is available to authorized users.

## Introduction

Archaeologists fancy ourselves as “time travelers,” and of course such an identity is at least implied, if highly problematically, in popular media. This attitude has certain paradoxical origins and effects, however, particularly in our own experience. When we encounter artifacts or features, it is of course them, and not us, that have “traveled” through time; we have merely managed to find them. Through our work and effort, however, we use them to create representations of what the past was like. When not sitting back proudly at all-encompassing representations of some long span of time, duly divided up into phases and temporal units, we tend to inhabit certain representations of particular points in time. We even inhabit certain points in the past to the extent that they dominate our views of other past moments or spans of time; we view other times from the vantage points of those conceptually more dominant moments and social worlds that occupied those moments.

This attitude has played out in our own “experiences” in our attempts to study the pre-Hispanic Tarascan State (ca. 1350–1530 AD) and the landscape of the Lake Pátzcuaro Basin (LPB) in central Michoacán, Mexico, in which the political and demographic core of that polity developed. The paradox of our experiences is epitomized by our problematic description of Jarácuaro (pre-Hispanic Xaraquaro) as the “ex-island.” Neither of us has directly perceived Jarácuaro as an island. Our mentor, Helen Pollard, has, and perhaps we carry on her memories as our own.^1^ More than this, we have also internalized the predominant practice, in itself entirely logical, of illustrating the lake level at the time of Spanish contact in 1520. We say logical because this is the point in time and the lake level at which the Tarascan State became known to Spanish conquistadors; in order to make sense of documentary evidence, it helps to “see” the lake and landscape as it was at this time. The practice of predominantly publishing images of the lake at this point in time, only beginning to change with data documenting lake fluctuations and particularly their relation to archaeologically documented past processes of inhabitation, has served to normalize this lake level in our minds-eye.

Problematically, however, this process of inhabiting (in the present) the basin as it existed very nearly 500 years ago also carries the implication of normalizing the existence of the Tarascan State. States and their development have long been a key problem in Mesoamerican studies and continue to be major contributions the culture area makes to comparative anthropology. These factors—normalizing the landscape of the state ca. 1520, the presence of the state, and the state as something to be investigated—have almost undoubtedly cemented the Late Postclassic period as the lens through which other periods are viewed. The preponderance of this lens, we argue, *prevents* a more sophisticated investigation of the processes that led to the consolidation of power known as the state. At the very least, the retrospective viewpoint from which we approach the state and the landscape that both affected its development and which it affected is problematic for reasons we detail in the course of this paper.

In contrast to this mode of normalizing and inhabiting a particular period, we must cast ourselves as temporal beings, just as past inhabitants of the basin were. Through archaeological investigation and assisted by new technologies, we are able to “inhabit” the basin (through representations) at various times and therefore “through time.” Van der Leeuw (1991; emphasis in original) has argued that archaeologists “will have to stop *looking back* from their present position in time, trying to recognize the past patterns that are observed in the present. *They will have to travel back* in time and *look forward* with those whom they study.” Adopting this viewpoint, Arnold (2008) prefers using the term “begoing” rather than “becoming” due to the presumption that what exactly something is becoming is implicitly imputed into the past. Finally, and to string these arguments together in the analysis of a more fully temporal mode of inhabiting, Sassaman (2012) has recently advocated for investigating alternative futures that were variously anticipated, planned, worked for, and/or unexpected. Of course, some of those potential future worlds were informed by memories of the past and knowledge of the present. It was the perception and formulation of such possibilities, through experience of presents and memories of the past, which shaped how peoples planned for, prepared for, and acted as agents of change in anticipation of what might come.

Forms of social action or agency that equate to managing time or controlling temporality have been recognized as essential in Western capitalist societies (Foucault 1971; Harvey 1990; Leone and Shackel 1987; Thompson 1967). On the other hand, temporality in the form of social change as well as periods of cultural reproduction and resistance have been linked to intimate perceptions of the landscape and an active social and temporal life of the landscape in many indigenous cultures of the Americas (e.g., Basso 1996; Dillehay 2014; Harris 1998, 2005; Nieves Zedeño *et al*. 2014; Sassaman 2012) and further afield (Hirsch 2006; Morphy 1995; Munn 1996). Sassaman (2012) makes explicit both the problem in drawing a strict distinction between the past and our present situation in terms of limiting the applicability of the past to the present as well as a view of some indigenous peoples of the Americas as somehow disconnected from time and temporality or only responding “after the fact” to ecological changes. Analyzing processes of state formation, as we are currently engaged in the LPB, of course has a long history of incorporating climate change, but often this is couched in terms of societies adapting systemically and only in the face of already existing adaptive pressures and ecological necessity as opposed to perceiving change, possibly anticipating it, and taking advantage of the opportunities offered by such change or alternatively preparing for its negative consequences. It is this possibility of investigating and envisioning processes of state formation as proactive rather than reactive, of investigating “past futures” (Sassaman 2012) in what was a highly dynamic environment, that this paper makes some initial steps toward realizing.

In this paper, we use geographic information system (GIS) modeling and representation built from various data sets, including archaeological research, in order to envision how past inhabitants of the LPB perceived time and temporal change in the landscape (and subsequently to analyze and discuss how they acted according to such change) and to integrate the insight of the “expanded now” (Gell 1992; Heidegger 1962; see below) within a framework based on intergenerational memory and the “eventfulness” of the landscape through time. Integrating those various data sets using GIS to reconstruct past landscapes and then using the ability of GIS to visually represent those landscapes, we analyze how pasts and presents would have, in any particular historical sequence and inter-generational period, affected how past inhabitants would have perceived their world and the range of possible futures that they could envision. We do this by discussing some aspects of phenomenological philosophy, particularly literature concerning “place” and “time consciousness.” Gell’s (1992) discussion of time consciousness as applied to the development of temporal maps leads us to formulate temporal charts of possible future worlds, much as Sassaman (2012) does. Finally, we apply Gell’s (1992) discussion of the formulation of the cognitive production of “time maps” to the advocated position of inhabiting the basin as past inhabitants did in a way that takes the importance of place and time more fully into consideration. In this approach, the landscape features of the basin, perceived and remembered in inter-generational time, became their own self-referential “time map” in which memories were materialized in particular features and even futurity could be gleaned from the 3D arrangement of the landscape. This “time map” and changes in it through time, moreover, provided its own framework for the interpretation of events, particularly what Whitington (2013) calls “bellwether” events. Such events, we argue, would likely have been perceived as spurs for social action and further landscape modification. First, however, it is necessary to discuss the nature of the basin, its fluctuation through time, and its role as the landscape upon which the Tarascan State developed.

## The Pátzcuaro Basin: Its Ecology and Role in the Formation of the Tarascan State

**Fig. 1:**
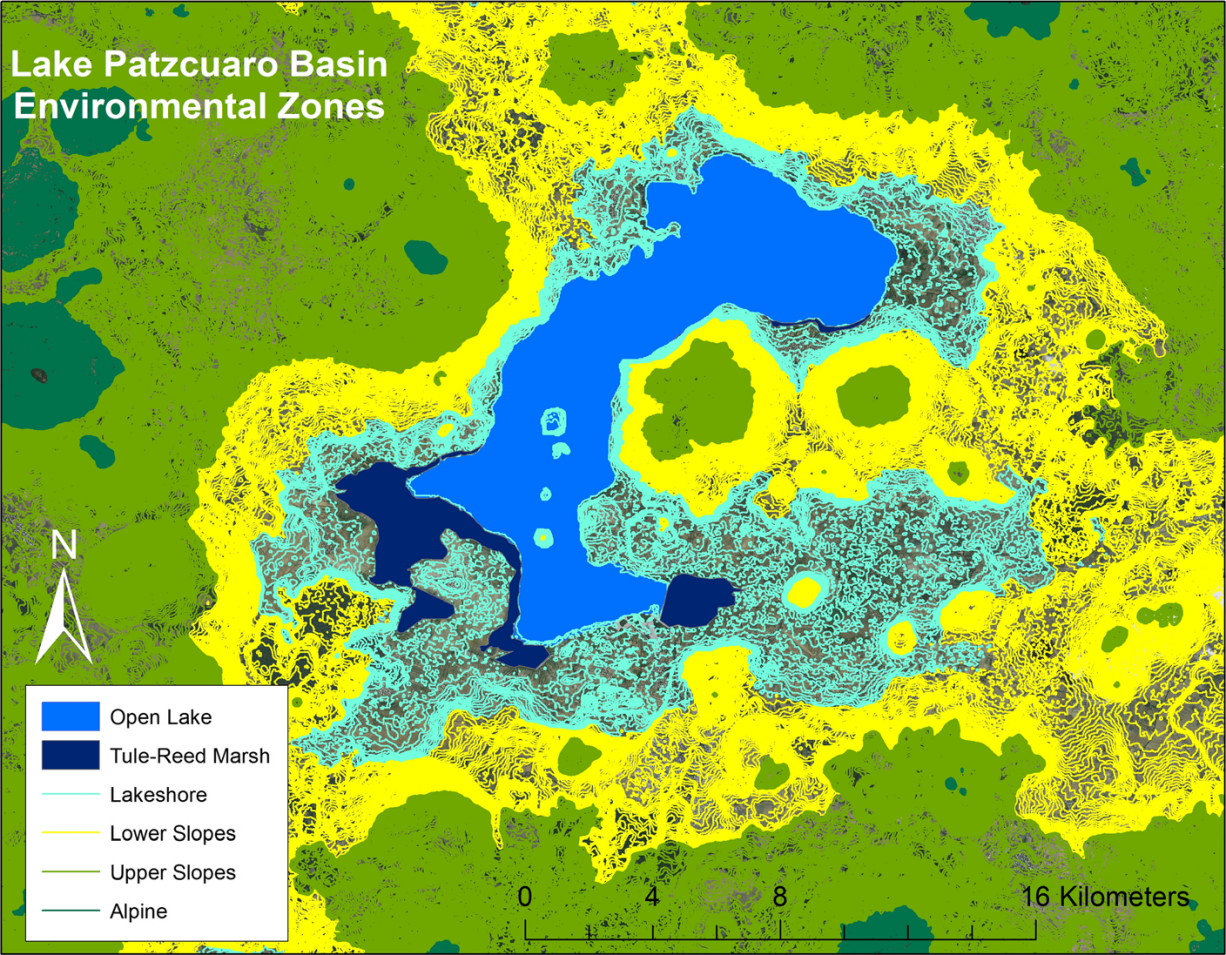
Resource zones of the Lake Pátzcuaro Basin with a lake level of 2028 masl

The LPB is a highland lake basin existing just above 2200 m above sea level (masl) and is ringed by varying amounts of gently sloping lands beyond which ascend mountains that range in height from 3000 to 3600 masl. This landscape provides numerous resource areas, including open waters that have been exploited for fishing and canoe transport, tule reed marsh (tule being used to make a number of goods, ranging from hats to mats), agricultural land of varying degrees of productive potential, and upland forested areas that supplied wood for fire and building materials (see Fig. 1). The lake level fluctuates about 0.5 m on average between the summer rainy season and the winter dry season. Larger fluctuations, due for the most part to climactic fluctuations and the fact that the basin is a deviation amplifying system, have been documented through limnological as well as archaeological data (Bradbury 2000; Fisher 2005; Fisher *et al*. 1999; Israde-Alcántara *et al*. 2005; Metcalfe and Davies 2007; Metcalfe *et al*. 2007; Pollard 2000, Pollard 2008).

These larger scale fluctuations and resultant changes in resource zones have been seen as an important factor in pre-Hispanic sociopolitical transformations, namely the emergence of the Tarascan State, in Pollard’s ecological approach (Pollard 1980, 1982, 2008; Gorenstein and Pollard 1983). Within this approach, increased hierarchy and complexity are interpreted to be adaptive to an ecological context in which foodstuffs necessary to support the population could be imported into the basin through a centralized tributary/administrative system that had been imposed through military conquests in the Late Postclassic Period, ca. 1350–1520 CE (Pollard 1980; Gorenstein and Pollard 1983). In later approaches grounded in models of ecological change and competition over scarce resources, Pollard (1982) proposed and then supported (Pollard 2008) with archaeological settlement data coupled with an increasingly detailed record of lake fluctuations (in part aided by said settlement data and archaeological studies of paleosoils (Fisher et al. 1999, 2003)) increasing scarcity of prime agricultural land that would have instigated competition and led ultimately to centralization in the transitions from the Early to Middle Postclassic and then the Middle Postclassic to Late Postclassic transition (see Table 1). Within such a model, the lengthy phases and the still poorly documented Middle Postclassic pose certain problems in interpreting exactly how and when elites and the larger population of the communities of the basin reacted to such circumstances and worked to shore up access to resources or gain the upper hand *vis-à-vis* rival communities. Interpretive dilemmas concerning the ethnohistoric record and its suitability for studying the pre-Hispanic past, as well as contrasting representations of the process of centralization itself, only further muddy the waters of investigating state formation (Haskell 2013).

**Table 1:** Period, phases, and recent lake levels that have aided in the reconstruction of past lake levels

Period	Phase	Year range	Year (modern/historic correlate)	Lake level (masl)
Late Preclassic to Early Classic	Loma Alta	100 BC–600 AD	2000 AD, 1999	2033, 2035
Middle Classic to Epiclassic	Jaracuaro, Lupe/La Joya	600 AD–900 AD	2000 AD	2035
Early Postclassic	Early Urichu	900 AD–1100 AD	2010 AD	2028–2030
Middle Postclassic	Late Urichu	1100 AD–1350 AD	2010 AD, 1973	2030, 2039
Late Postclassic	Tariacuri	1350 AD–1525 AD	1940 AD, estimated 2043 level	2041, 2043

This socio-natural history of lake level fluctuations along with settlement pattern expansion and transformation (Pollard 2008; Stawski 2012; see below) can, from Processualist and adaptationist theoretical viewpoints, be interpreted unproblematically as “giving rise” to the state and “selecting for” greater sociopolitical integration and the need to import foodstuffs for a population that is growing even as critical landscape zones are shrinking. Such an interpretation suffers key weaknesses, however, that a phenomenological approach can begin to rectify. First, the above interpretation is teleological—it imputes into the pre-state era actions that would, as far as we know, only be necessary in the Late Postclassic as population continued to expand. This contradicts the emerging view that the Middle Postclassic is when the earliest steps toward political centralization appear to have taken shape (Pollard 2008, 2015). Second, this teleology only serves, once again, to naturalize the state and to cast its development as the necessary outcome of population pressures. Third is the concomitant point that nowhere in this picture are actions and strategies of actual people in the past; where/when people are present it is only in an adaptive and reactive mode (again such reaction goes against the view that the state has its roots in the Middle Postclassic; Brumfiel (1992) prominently called attention to the need to investigate processes and competition within societies in opposition to systemic models). The total effect is to fail to investigate what actually happened and why in terms of the experiences and agencies of the people that produced this transformation. At the same time, by naturalizing the state, this teleology fails to explain why resource competition (resource scarcity or the population outpacing the productive capacity of the agricultural regimes apparently did not occur until the Late Postclassic) did not result in some outcome *other* than the state; we contend that the Tarascan State is only truly analyzable in terms of the agency and strategies, manifested in and through time, of peoples and communities. Furthermore, phenomenology aids us in investigating these agencies and strategies because it situates them within a landscape and temporal context in which the perception of landscape change and the ability to plan for unpredictability, and to paraphrase Sassaman (2012) and Koselleck (2004) to act in order to increase the likelihood of some futures while mitigating against undesirable potential futures, likely made a difference in the processes of state formation.

GIS has been used to represent and model the interrelationship between the lake level stability (or lack thereof) and reorganization of the settlement pattern and the relations between the two (Stawski 2012). What GIS has not been used for, either in the LPB or in general terms for other changing archaeological landscapes, is to render the landscape through time and to therefore perceive *through time* the changes that past inhabitants experienced, thus casting the landscape and the state that called it home during the Late Postclassic as contingent rather than given, but also as the subject of present and future temporally manifested projects within the flux of time in the past (following Koselleck 2004; Sassaman 2012). To do so requires bringing together a range of limnological, historical, and archaeological studies in an integrated fashion as well as the contributions and perspectives of phenomenological philosophy.

## Constructing GIS models of the LPB from the Classic to the Late Postclassic Periods: a Baseline for Analyzing Socio-Natural Dynamics and a Phenomenology of Past Futures

The method for landscape reconstruction, performed by Stawski (2012), began with two data sets. The first data set derives from geoarchaeological, archaeological, paleoeocological, and geological work in the region, which analyzed and approximated the pre-Hispanic lake levels for the temporal sequence in the LPB. Such data includes the Watts and Bradbury core, or “Master Core” as it is called, which was taken in 1973 and was 1520 cm in length and dates back to the Pleistocene; it is approximately 44,000 years old at the base (Watts and Bradbury 1982, p. 56; Bradbury 2000). Since that initial core, others have been taken and reported on in various parts of the lake and adjacent area. Fisher (2000), Israde-Alcántara *et al*. (2005), Metcalfe and Davies (2007), O’Hara (1993) and O'Hara *et al.* (1993) all report on cores, trenches, agricultural wells, and exposed cross sections to construct the sedimentation record, its chronology, and explanations for the deposition of certain sediments during certain time periods. Archaeological data for the pre-Hispanic lake levels derive from Pollard’s long history of research in the LPB which is synthesized in her 2008 publication.

The second data set derives from a series of historic and modern aerial remotely sensed images of the LPB, ranging from the 1940s to 2010. Since the 1940s, Lake Pátzcuaro has undergone very similar change, with regards to lake level and environmental zone fluctuations, to that of the change documented for the pre-Hispanic sequence. As a whole, the landscape is just as dynamic as the lake, and yet certain aspects remain relatively stable. It is the view of Watts and Bradbury, whose main cores aided in reconstructing flora, climate, lake, and sediment changes for the basin since the Pleistocene, that “the character of the vegetation surrounding Lake Pátzcuaro has not changed drastically in the last 40,000 years” (1982, p. 59). This allows us, therefore, to reconstruct the pre-Hispanic landscape through the use of historic and modern data for the period of pre-Hispanic occupation of the LPB.

The imagery, which was accessed through the USGS imagery repository, included imagery from the various Landsat satellites (1973, 1976, 1979, 1980, 1989, 1996, 1999, 2004), ETM Pan Mosaic Imagery (2000), and the Tri-Decadal Global Landsat Orthorectified TM Mosaics (1984–1997). The most recent imagery (2010) was acquired from the SPOT satellite and is a rectified SPOTMap mosaic of the entire LPB and coupled with a SPOTMap digital elevation model (DEM) was the basis for creation of 20-, 10-, and 5-m contour maps, slope maps, and 3D imagery of the landscape. The earliest imagery, from 1940, consists of aerial imagery taken by the US Army Air Corps and declassified after World War II. This imagery was scanned, digitized, georeferenced, and rectified in ArcGIS. All imagery, if not so in the original form, was standardized through georeferenced and orthorectification to WGS_1984_UTM_Zone 14N. These aerial images cover the past 70 years of lake fluctuations of the LPB, from lake level elevations of 2041 to 2028 masl. Through the use of the ArcGis suite of tools, environmental zones in the lake basin were accurately located and measured from the aerial images. The specific method used for this derives from Hritz (2010), Gomez-Tagle Chavez *et al*. (2002), and Brivio *et al*. (2000), and it should be noted that this method not only used visual inspection to locate environmental zones but also relied on certain wavelength signatures found in the color satellite images to locate certain environmental zones based on vegetation reflection.

The aerial imagery was analyzed in GIS, and for each unique aerial image, shape files were created for the open water, tule-reed marsh zone, islands, and the lake shore. For example, the lake boundary was traced for each aerial image representing a specific lake elevation at a given year. This was repeated for each aerial image in the collection from 1940 to present. The outcome was a series of lake shapefiles in vector format, representing the various fluctuations Lake Pátzcuaro has undergone since the mid-twentieth century. Once the shapefiles are drawn, each can be measured in size, thus creating a layer of data that can be analyzed in terms of what percentage of the total basin each environmental zone comprises.

These newly created shapefiles represent a specific lake level elevation and the corresponding environmental zones related to that lake elevation. The modern lake levels and the corresponding GIS layers were then correlated with the pre-Hispanic lake levels that had been estimated and reconstructed from the first data set, allowing for a pre-Hispanic reconstruction of the lake elevations, lakeshore, the marsh zone, and the islands. Table 1 shows the correlations.

The next step was to reconstruct the other resource zones of the basin. Gorenstein and Pollard’s (1983) assessment of the land classes and the environmental zones was the guiding data, which were collected from ethnographic, ethnohistoric, aerial, and field reconnaissance. There are six major environmental zones: (1) the open water zone, (2) the tule-reed marsh, (3) the lakeshore, (4) the lower slopes of the sierra, (5) the upper slopes of the sierra, and the (6) alpine.

Gorenstein and Pollard (1983) also classified the landscape according to the extensive geological, geomorphological, soil, and climatic data and supported further by ethnographic data, which was used to determine agricultural practices in the basin during the first half of the twentieth century. The class I land consists of that land which is permanently watered, by “either canal or pot/ditch techniques,” and seasonally watered, in which the “land is under seasonal irrigation by flood water techniques.” Class II land consists of “land in the flattish floor of the basin (Lakeshore environmental zone) and the alluvial basins of the Upper Slopes environmental zone,” which is farmed by rainfall agriculture. Finally, class III land includes all the remaining agricultural land in the basin, including areas of the lower and upper slope environmental zones, forest, pasture, the tule-reed marsh, and open water. This data provides information vital to the analysis of the terrain, slope, and elevation of the landscape, which was reconstructed using a 3D DEM in GIS, the imagery having derived from the SPOT satellite^2^.

The resulting GIS data sets are compilations of shapefiles that accurately depict certain temporal and spatial stages of the landscape with the pre-Hispanic past. By envisioning this landscape through the eyes of the past inhabitants, using viewsheds from various vantage points that are easily constructed thanks to GIS, we can begin to understand the dynamic human-environment relationships that occurred and how it shaped perceptions of lived time-space. The basic technique of viewshed operates on a DEM to determine which areas are visible from a given 3D location (Van Leusen 1999, p. 218). In archaeological research, viewsheds have been used widely to elucidate factors that may influence settlement, monument location, and resource allocation, to name a few (Jones 2006, p. 525). In the case of the LPB, the SPOTMap DEM, a high-resolution digital terrain model, was able to produce an accurate 3D model of the lake basin, on which the reconstructed vector images were overlaid, thus producing a 3D reconstruction of the landscape or what Llobera (2003) refers to as a *visualscape* (see images 3a–3f). Furthermore, this method was replicated for each “snapshot” of the LPB during each pre-Hispanic phase, thus producing a time-lapse of the fluctuations and changing resource and environmental zones for the entire sequence. In essence, this shifts our static conception of the landscape toward something much more dynamic and temporally constituted.

The “landscape” was not simply the natural environment; this term implies human perception, inhabitation, and contestation (Ashmore and Knapp 1999; Bender 1993; Ingold 1993). In this regard, archaeological evidence helps us understand the long-term history of human inhabitation of the LPB. From the earliest well-documented settlement patterns in the Classic period, settlements in the LPB were primarily oriented to the lake and its resources (Stawski 2012; Pollard 2008), as the lake level was stable during this time (O’Hara *et al*. 1993). The Epi-Classic and Early Postclassic saw a drop in lake levels, the inhabitation of newly exposed lands, e.g., low *lomas* around Jaracuaro (Pollard 2000), and in general the continuation of lake-oriented settlements. This lake regression was followed immediately in the Middle Postclassic by a transgression event that continued through the Late Postclassic period. Settlement patterns shifted away from the lake for the first time in the pre-Hispanic sequence and focused on slightly upland towns and especially nucleated administrative, market, and religious centers (Pollard 2008; Stawski 2012). Agricultural terraces have been documented that, due to their presence upslope from Late Postclassic population centers associated with those settlements, are most likely associated with those centers (Fisher 2005), and we return to the significance of such landscape modifications below.

## Landscape and Place in Phenomenological Philosophy: Experience, Practice, and Dwelling in the Pátzcuaro Basin

In phenomenology, as a philosophy of how humans experience and are relationally bound up with places and things, places are not mere spatial entities but gatherings of other inter-related places, with this gathering mediated by human experiences and meanings. In analyzing the experience of the changes of the landscape, Heidegger’s (1962) concept of “tools” is enlightening, though we emphasize adding a twist to that concept. In Heidegger’s discussion, human interaction with the world is mostly concerned with actively using tools (i.e., anything involved in productive action). In such interactions, the tool simply “works” and becomes intermeshed within the project in which it is involved. Heidegger calls this “ready-to-hand.” When functional, and “ready-to-hand,” it is in its proper state of being, working, affecting the world, and being entangled in any multitude of relations in its being. In opposition to this aspect of being is “present-at-hand.” The “presence” of the object, e.g., a hammer in Heidegger’s own analysis, only arises when it is made the subject of reflection and abstraction. Only when the hammer, as part of the overall “equipment” involved in any task, fails us do we think reflectively about the hammer as one particular object. Individual objects only become isolated and isolatable in this occurrence and in this manner, but we lose the active possibilities of everything that the being of the hammer offers. In “presence-at-hand,” objects become divorced from the myriad or even endless possibilities that their being might effect on the world and becomes an inert and singled-out object.

If we think of past inhabitants of the LPB and the features, resource zones, etc. being intertwined and used as relationally entangled “tools” *over time* and within these fluctuations (and following Ingold’s (1993) discussion of the “taskscape”), Heidegger’s analysis and disjunction become problematic. Within such constant change (remember that even in periods of lake level stability the lake would have still fluctuated seasonally), we can imagine the overall equipmental system “lighting up” and bursting into “present-at-hand” reflective consciousness *all the time*, even as they remained enmeshed with other places. To a large extent, then, we suggest that Heidegger’s dichotomy is overwrought and that past inhabitants of the LPB would have simultaneously perceived the regressions and transgressions that brought specific places into conscious reflection but remained enmeshed in practical projects within the overall taskscape. In the larger picture then, past inhabitants would have been highly cognizant of its dynamic nature and likely had a number of practices and schemas through which the importance of the lake’s dynamism became part of their cultural matrix.^3^

In concordance with this eruption into reflection, philosophers and anthropologists drawing on phenomenological roots have discussed how within human perception and interactions with “place” as opposed to Cartesian “space” (Casey 1996; also Basso 1996; de Certeau 1984 reverses the use of place and space, but the distinction is the same), places are “events.” Similarly, Olsen (2010) discusses “things” *not as objects but strung-together events* within and as a result of Heidegger’s philosophy. Places happen and possess their own temporal characteristics. In order to fully account for “place,” then, we must examine the relationality of place not only in spatial terms (other places in the midst are gathered and serve to comprise any one place) but also in temporal terms. A place is, at any one time, the sum of its recollected and, as we discuss below, planned-for events and the social and landscape entanglements of those events.

This point can be further strengthened by additional insights from phenomenological philosophy as argued by Harman (2002, pp. 61–66) and the work of Gell (1992). The relationality of time is already present in Husserl’s description of the “extended now” created by retentions and protentions (see, e.g., Gell 1992, pp. 221–241). The “extended now” is also present in Heidegger and Henri Bergson, for whom “time cannot be viewed as a sequence of now-points” (Harman 2002, p. 65). As we understand it, any instant cannot be isolated because the “meaning” of that instant can only be defined according to its temporal relations. An instant at which the lake level is 2035 masl that is surrounded by other instants at which the lake is also at 2035 masl is a completely different kind of instant than if it had been surrounded by other instants in which the lake was at multiple different levels.

The perception of place and its eventfulness occurs, just as changes in the landscape, at various scales. Within the taskscape, places gather daily activities, roles within seasonal rounds, and as places were transformed through larger temporal transformations such as appearance or disappearance, expansion or retraction, social practices and labor investment, etc. These scales interpenetrate one another; daily rounds heighten awareness to the landscape and attune attention to its larger scale fluctuations and their “eventfulness.” Furthermore, Merleau-Ponty (1962) points out that inhabitation of the landscape is always an embodied one. Therefore, perception and cognition are grounded in the body and its movement, in particular by the feet and through walking (Ingold 2004). Given the geography of the LPB, we hasten to add other forms of movement, particularly canoe-based travel, and its equipment (canoes, oars, fish and fishing nets that give these objects part of their reason for existence, other goods that were transported across the lake, and of course water). Each form of movement existed variously in coordination or in opposition to each other according to changes in the landscape; lake transgression necessitated canoe travel and rendered walking impossible in certain places. This necessarily implies that, for the most part, perception was terrestrially bound, as opposed to perceiving the landscape from above as in the form of maps (a point made by de Certeau (1984)).

Envisioning the landscape and inhabiting it through terrestrial movement and temporally dictated tasks would have contributed to the sort of collective memory that Connerton (1989) categorizes as being sedimented and passed on as an incorporating practice. Memory is incorporated into the integrated mind-body through routinized or regular practices. When we imagine “communities of practice” (Lave and Wenger 1991; see also Harris 1998, 2005 on place-based and historically constituted enskillment) that inhabited the LPB over successive generations, therefore, we can easily envision experienced elders passing on to neophytes information regarding the land, the water, the extent of resource zones, and most importantly how these have all changed within their lifetimes (akin to our own introduction to the basin and its changing nature). We must however recognize a potential or hypothetical (i.e., which more data, particularly ethnographic data, can further evaluate) limit to this process of passing down remembrances. Following others (Sassaman 2012), we believe a 50- to 100-year limit, based on intergenerational memory and transmission of remembering such data, is an appropriate starting point. In this way, a neophyte might learn from a parent or grandparent what that family member had heard from their parent or grandparent concerning landscape changes.

Communities of practice existing through time and passing on generational knowledge allow us to discuss the likely anticipation of various conditions in the future that are longer than individual “extended now moments,” shorter than the centuries-long archaeological phases, and, because of their continual nature, bridge across the perceptual chasms between such phases. Such memories can be easily fit into the experience of time developed by Husserl in which past events are perceptually held in the present as retentions (see Gell 1992, pp. 221–228). Similarly, retentions from the past as well as present experiences have meaning in relation to protended or anticipated future events (see also Sassaman 2012).

## Mapping Time in the Landscape: Gell and “Time Maps” in the Lake Pátzcuaro Basin

One final discussion is necessary before we can address the past landscapes of the LPB and how past peoples inhabited them. This is Gell’s (1992, pp. 149–174, 237–260) development of an anthropologically informed means of relating what is called A-series and B-series time. Beyond the “expanded now” delineated by Husserl (Gell 1992; Lucas 2005), humans experience time within a “past-present-future” framework in which time or events seem to come at us from the future, occur in the present, and slip into the past. This is “A-series” time according to McTaggart’s original formulation (Gell 1992; Lucas 2005) and is also known as “subjective” time. “B-series” time, on the other hand, can be categorized as “objective” time insofar as within the B-series, events are dated and such dates do not change. If event *E* occurs at time *T*, it will always have this property, as opposed to *E* being variously in the future, present, or past within the subjective time of the A-series. While we cannot directly experience or perceive the *totality* of the B-series, we nonetheless make cognitive maps of the B-series based on our A-series experiences, memories, etc. (Gell 1992). Out of the baseline subjective classifications of past, present, and future, we can order different events and construct a sequence in which all events are related to all others and occupy a fixed point within the time map. Gell likens this process to constructing a map of space out of the various subjective experiences of perceiving places and traveling between them, a metaphor whose distinction between subjective paths and totalizing space is quite germane to the discussion below of using the LPB as a time map.

Gell also makes use of the experience of time within retained memories and protended anticipations to construct a diagram of possible worlds based on such retentions which could be protended into the future. As we construct time maps of things long-past as well as more recent events, events within an “extended present,” and in the future, we place them at different distances from “now” accordingly. Various distances also imply varying degrees of possibility in terms of assessing the likelihood that something like a remembered or passed down event will happen once again (see Gell (1992) figure 25.3; such distances and the likelihood versus improbability of places at various distances happening also leads to Whitington’s (2013) discussion of “bellwether” events discussed below). Using Gell’s insights and combining them with a relational and place-event approach to envisioning the landscape of the LPB, we can use the individual features of that landscape to envision time in the landscape and as its eventfulness was perceived by past inhabitants. We can now proceed to examining geographic and archaeological settlement data to appreciate the effects of this perspective on interpreting actual past temporalities and lived lives. Through this data, we investigate a certain way of seeing time *in* the landscape and mentally mapping temporal changes through the continual “happening” of place and the placed-based eruption of time/place reflective consciousness produced by seasonal fluctuations, regressions, transgressions, and the social practices that were bound up in the landscape and these events.

## Inhabiting the Past and Constructing Time Maps in the Lake Pátzcuaro Basin

GIS is useful for helping us envision and begin to dwell in the landscapes of the past. Viewshed images produced using the DEM have the advantage of allowing us to perceive the landscape as an embodied subject would, from the ground, as discussed above. Vertical shifts in water would also affect the various landforms and environmental zones in their horizontality, either exposing or covering islands, the tule-reed marsh, the lakeshore, and the lower slopes. Within these viewsheds and the perception of a changing landscape full of tasks, it is easy to imagine the constant calculations of changes in the time allocation (as in the approach of Hägerstrand 1973; see also Gell 1992, pp. 190–197; Pred 1977) required when various resource zones are further, closer, or perhaps even newly non-existent or non-accessible as well as newly existing and accessible. This is the second manner in which GIS can help envision the changing landscape and humans’ past interactions with it, by computing changing distances to various resources. For example, even within a seasonal fluctuation of half of a meter in elevation, the lakeshore “boundary” fluctuates an average distance of 130 m, sometimes flooding previously dry land, and in some cases exposing lakebed bottom as new arable land. Such perception is also bound up not only in the tasks that must be performed but includes the burdens that must be born across the landscape as part of those tasks (Cuelenaere 2011), as in Hirshman and Stawski’s (2013) discussion of potters transporting their wares to market in the LPB. In that discussion, Hirshman and Stawski focus on terrestrial transportation, but various changes in the landscape perhaps made such journeys alternatively easier or harder (as in the change of solid ground into marshland or vice versa) or possibly forced canoe-based transport in certain instances.^4^

Examining these viewshed images enables us to envision the landscape as it was changing, in a rough approximation of what past inhabitants would have experienced. Through this data, we investigate a certain way of seeing time in the landscape and mentally mapping temporal changes through the continual “happening” of place produced by seasonal fluctuations, regressions, transgressions, and the social practices that were bound up in the landscape and these events. In relation to the recollections of previous generations that were passed down, these kinds of perceptions of the landscape would have served as a spatiotemporal means of organizing these recollections and perceptions. Organized as a spatial map of landscape features that variously “happened” in daily practice and their “happening” at larger temporal scales due to lake level changes and in relation to their unique characteristics, the locations of these landscape features as well as their elevations would have constituted each feature as a sort of encapsulation or indication of a particular time and the lake level at that time as well as the extent of resource zones. As such, they would also have functioned as indications or bellwethers of changes that have already taken place and, given the horizontal movement of the lake, changes that might be yet to come (Whitington 2013; on “time indication,” see Lucas 2005). In this way, the landscape was “self-referential” because not only did these landscape features serve as indexes of specific lake levels in their vertical variation but their very horizontal relations and spatiality demonstrated the implications of such lake levels in terms of the spatial extents of resource zones at those indexed lake levels. The appearance and disappearance of features such as these likely signaled that the landscape was changing in ways not experienced before and instigated reflection on the numerous consequences of such events. Furthermore, these changes would have been significant for certain segments of the population that either lived on or depended on such features for their livelihood. One example is the evidence recovered by Pollard’s (2000) survey of the southwestern part of the LPB of residential occupation on the small *lomas* surrounding Jarácuaro in the Early Postclassic that would have had to be abandoned (and indeed were abandoned, as archaeological evidence indicates that these were single-phase sites) as lake levels rose.

**Fig. 2:**
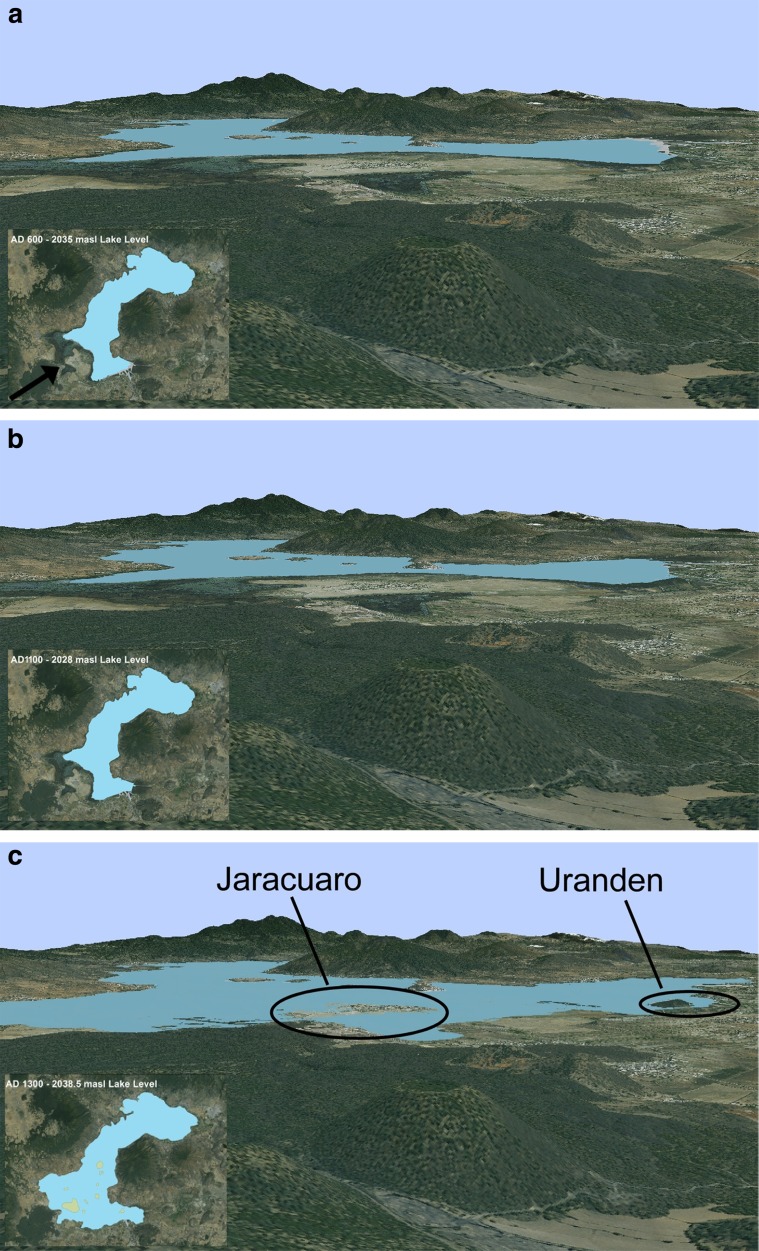

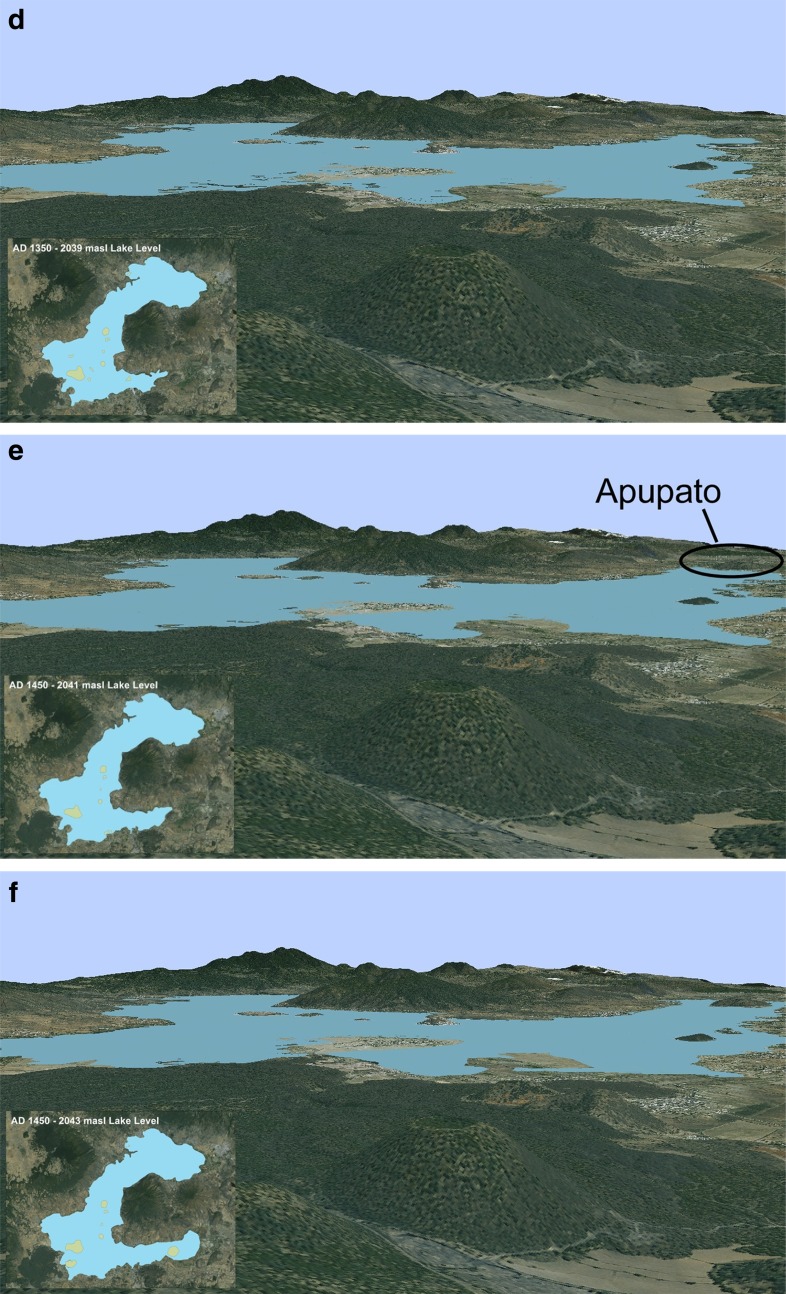
**a**–**f** Viewshed of the Lake Pátzcuaro Basin looking northeast (see arrow in Fig. 2a) at various points in the pre-Hispanic era. The approximate year is given in the reconstructed landscape as viewed from above in the lower left of each figure

Viewshed images (Fig. 2a–f) of reconstructed landscapes of the LPB as seen from an elevated position at the site of Urichu in the southwest basin, at various points in the past illustrate these points. For considerations of space, we include only those images pertaining to 1100 CE–1500 CE; earlier eras were fairly stable in terms of lake level (see Table 1). We suggest the best way to views these pictures and to “inhabit” them “temporally” is to study them closely and identify all of the various landscape features that change, particularly “islandification” events, as landscape features become islands or the reverse—are united with the mainland. To construct just one (fairly coarse grained) “time-place map,” Jarácuaro becomes an island roughly coeval with one Uranden, and the next Uranden becomes an island at about the same time the lake stretches east and touches the slopes of Apúpato, then the landscape feature south of Jarácuaro shrinks drastically as the lake stretches southward at about the same time it also isolates another of the Urandenis and stretches eastward to the western base of Apúpato, the Urandenis become islands later, and finally the feature south of Jarácuaro (Copujo) becomes an island at the same time as Apúpato. Meanwhile, numerous slight *lomas* in the lake area between Jarácuaro, Erongarícuaro, and Urichu come into and out of existence, their number and location indicating lake levels and the general direction of slightly up-or-down-sloping land. All of these changes would have radically affected seasonal if not daily practices of acquiring resources, transportation, and farming, and perhaps also including negotiating access to resource areas, in particular good agricultural land. Such negotiations and contestations would have been necessary possibly in the short term and almost assuredly in the long term as the lake level regressed in the Epi-Classic and Early Postclassic and then particularly in the transgression that spanned the Middle and Late Postclassic periods.

## Remembered Pasts, Perceived Presents, and Anticipated Futures

In this way, we can at least begin to envision the construction of time maps to represent the significance of lake regression and transgression processes and the place-based “events” that they instigated in relation to one another. Such recognition is made directly relevant to human planning and agency by envisioning how these changes in the landscape would affect the anticipation of yet further, future changes to the landscape. After all, if the landscape had changed in the past, it was likely to change in the future; moreover, perhaps the changes that had been witnessed in the past and were being witnessed in the present could hold clues for what should be expected for the future. In this sense, using the landscape of the basin as a time map of potential worlds can help us understand the nature of dwelling and active planning for the future on the part of past inhabitants of the LPB. The various landscape features depicted in the reconstructions and described above would have led to the ability to remember and pass down fairly specific information regarding past lake levels and thus to implicate those levels as possibilities in the future.

**Fig. 3:**
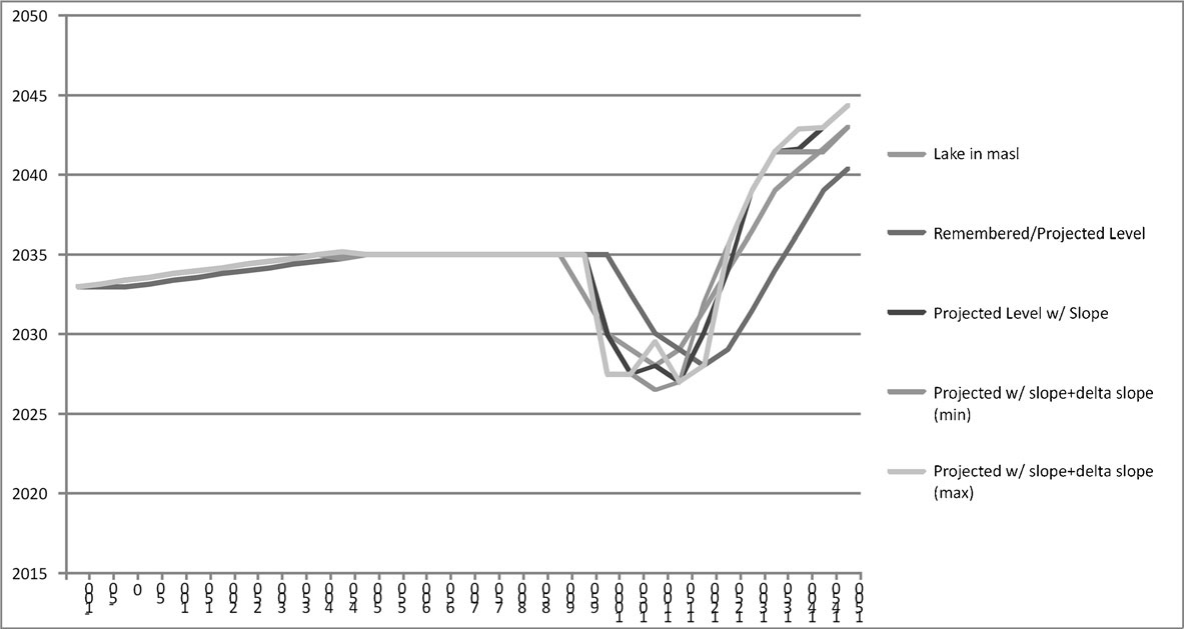
Graph of actual and possible lake levels given remembered and protended levels, as well as projections of lake levels that take into account past changes (slope) and rates of change (delta slope), all calculated at 50-year intervals. The maximal space between the various lines would have been the possible landscapes that inhabitants of the basin likely thought possible in their lifetimes

The result, Fig. 3, incorporates Gell’s (1992, Fig. 25.3) diagram and its “envelope of action” with some of Sassaman’s (2012) insights concerning how to diagram change in the experience of past inhabitants of a region and apply them to the context of the LPB and its fluctuations as they would have been experienced, remembered, and used to envision and anticipate alternative futures. The individual lines of Fig. 3 show the lake level, the same lake level remembered/passed down 50 years and then protended 50 years into the future, the projection of current rates of change (the slope) 50 years into the future, remembered changes in the rates of change (delta, both in terms of absolute value and either positive or negative) applied to the currently perceived slope and the resulting lake level projected 50 years. We believe the 50-year interval between remembrances and protensions is fairly conservative, particularly considering the place-based “eventfulness” of the LPB landscape, as discussed above. The maximal area between the lines can be conceptualized as the potential worlds (lake levels) that could be reasonably projected based on memories of changes received from the past. Note how in the Postclassic, from roughly 900 AD until contact, the gap between the lines is at its maximum, meaning that the widest range of future worlds could be protended during these years. While lake level fluctuations during the Postclassic appear dramatic in terms of the time resolution of archaeological phases, when viewed in human intergenerational time they would not have been sudden. We believe that this indicates that protended worlds based on memory were not so massive and unforeseen that they could not be anticipated and planned for, a point to which we return later.

To tie this graph to the viewshed reconstructions, two examples using the images from Fig. 2 are illustrative. At ca. 1250 AD, the lake was at 2036 masl and therefore the landscape looked like it does in Fig. 2a. Taking into account perception of the rapid change of the lake transgression event and as identified in the graph, however, it might have been reasonable to imagine that at sometime soon the lake would be anywhere between 2030 and 2038 masl. Those inhabitants would have been able to imagine the landscape looking approximately like it does in Fig. 2b and c. A century later in ca. 1350 AD, the lake was at about 2040 masl—the lake and landscape would look like something between Fig. 2d (2039 masl) and e (2041 masl). Inhabitants would have been able to imagine that in the very near future, given lake level changes, the landscape might range from how it looks in Fig. 2a (at 2036 masl) to f (at 2043 masl). Note furthermore that while lake level changes at lower lake levels certainly would have affected the amount of exposed or inundated land for certain settlements (especially areas northwest of Jaracuaro), the landscape takes on a new degree of “eventfulness” in terms of landscape features becoming islands and the loss of land at higher lake levels and at later times. This is the case especially in its southern and eastern areas thanks to the elevation and topography in those areas; the landscape reconstructions and viewsheds allow us to see that lake level change by itself, for example at lower lake levels in the Early Postclassic, does not necessarily equal eventfulness. It is only as specific lake level changes meet the topography of the basin as perceived and experienced by inhabitants that landscape events happen.

## Envisioning Time and Place and Mobilizing Social Action

It is essential to note that lake changes, and resultant transformations of the landscape, were not the only relevant changes occurring among human-environment relationships. Populations grew, slowly at first in the Loma Alta phase but then more rapidly, particularly beginning in the Early Urichu Phase (as the lake was receding and exposing more land). Social hierarchies emerged in the Loma Alta and became more marked as recognizable hereditary elites emerged, sometime around the Lupe/La Joya phases and definitively by the Early Urichu phase (Pollard 2008). Most likely, there was some accretion (and abandonment) of landscape modifications through the years, particularly the terracing (and its after-effects) studied by Fisher (2005). At the same time, economic behavior remained largely local in scope (Pollard 2015) and settlement patterns remain organized around the lake’s margins for centuries (Stawski 2012).

In relation to the above practices and characterizations, Dillehay’s (2014) work leads archaeologists to consider the ways in which power influences the processes whereby perception and knowledge are either democratized or restricted and manipulated—in other words, differences of perception constitute political projects and are recursively engaged with power differentials such that it springs from such differentials and helps reinforce them. We should not equate phenomenological theory with a naive process of “being there” in which the problematic nature of both “being” and “there” is left unaddressed or left to exist apart from considerations of power. Rather, we must attempt to investigate how differential power relations and modes of sociality affected perception. Therefore, it is necessary to at least discuss how restricted or widespread such perception and knowledge might have been through time, particularly if members of the elite took political advantage of such shifts and their actual or likely consequences. We cannot determine if certain lands were forbidden from various segments of communities, but we can discuss what must have been typical practices that informed perception. Drawing on communities of practice (discussed above) and subsistence/economic patterns, interpretations of ethnohistoric data, and ethnohistoric and archaeologically documented information concerning marketing and the potential for non-elite modes of information sharing help us begin to address these concerns. By discussing the practices by which various segments of communities in Michoacán perceived and conceptualized the changing landscape and the concomitant formulation of potential consequences and advantages/disadvantages therein, we can better formulate how elite calculations of alternative futures drove state formation but in a way in which commoner knowledge was essential and they were not mere targets of elite manipulation and machination but significant components in community processes and projects in their own right. State formation would have involved both the mobilization of labor and organized violence among the populaces of various communities, grounded in their daily experience and perception (much as in Dillehay’s (2014) example), but these were constituted by the perception of change and the representation of futures in which whole communities participated, but differentially so.

Perception among the bulk of the commoners would be bound mostly to lower elevations in the LPB. Fishers, farmers, and craftsmen would have been engaged in daily practices that did not involve substantial movements to higher elevations. The transportation of goods to market would have added another itinerary to the extractive and productive rounds of such commoners. The presence of markets as places where producers could gather and share information with members from other communities is an important question that fellow archaeologists (Gorenstein and Pollard 1983; Hirshman and Ferguson 2012; Hirshman and Stawski 2013) are beginning to address. The presence of a fairly consistent range of pastes in ceramic samples at the sites of Urichu, Erongaricuaro, Jaracuaro, and Pareo reveal some changes but overall can be characterized as the result of a durable and predominantly localized marketing practices in which goods changed hands most frequently between people of the same areas of the basin or at the very most people within the basin. Within such localized marketing behavior, and in the most comprehensively studied portion of the LPB in which the above sites are located, the market at Pareo at a lower elevation would not have necessitated travel to higher elevations. As best we can surmise, then, most commoners would not have been involved in travel to higher elevations that would have facilitated the perception of the landscape from above as in the perception of spatial maps, instead being largely restricted to their own spatial stories (following de Certeau 1984). Changes in the landscape would have been perceived, depending on the particular commoners and their particular rounds and spatial stories, but we suggest that these stories would not have approached more totalizing perceptions of the landscape, its changes, and more so the full impact of its multiple potential futures.

Foreign goods such as Pachuca obsidian during the Classic period and artifacts of Central Mexican “Toltec” style (particularly ceramic, including flutes found in a burial at Urichu: see Pollard and Cahue 1999) in the Early Postclassic did find their way into the LPB, suggesting some amount of long distance trade and merchants that traveled between regions. These trade goods are limited to the political elite during the Postclassic (and perhaps a burgeoning elite or kin unit leaders in the Classic period). This indicates that elites would have had certain knowledge, either firsthand or secondhand from the long distance (or at least more regionally oriented) merchant contacts by which such goods flowed into the LPB. It is an open question if commoners would have interacted much with these non-local long merchants in the marketplace and thus if they would have received descriptions of the changing landscape from these merchants that would have had to cover higher terrain in their travels.

Woodcutters would have had business in the higher elevations and would have likely been commoners that interacted with other commoners—lumber was simultaneously used for fires, as an important construction material (highland Michoacán is still famous today for its finely crafted wooden architecture), and as an offering to the gods. While the last of these economic roles implicated elites as well as commoners who would have been required to offer it as tribute (Alcalá 2000), the need to cut wood for common cooking fires and dwellings likely meant that commoners also supplied wood to the communities of the LPB. A potting industry consisting mainly of commoners and spread across the landscape of the LPB (Hirshman and Ferguson 2012; Hirshman *et al*. 2010) would have also required wood for firing their wares. Woodcutters would have had some ability to perceive the landscape in a more totalizing fashion and been able to share that perception with other segments of commoner society. The market at Erongaricuaro in the early colonial period into modern times, for example, was a place at which wood was regularly traded for lake products (Gorenstein and Pollard 1983, p. 52; West 1948). West’s (1948, plate 14) ethnographic photographs record a firewood and fish market at Erongaricuaro and a canoe from the island of Janitzio landing at Erongaricuaro for the market there in the 1940s. Such practices at pre-Hispanic markets would have likely been nearly identical and involved similar segments of society. It is worth noting that González (2010) posits routinized ritual exchanges and communal festivals organized by and composed of segments occupying complementary ecological niches, such as lakeshore and upland forests.

In sum, a full consideration of movements and perceptions indicates that the majority of commoners would have been highly cognizant that the landscape was changing, but that cognition and reflection would have been quite local in scope, at most augmented by woodcutters. Such changes, significant in impact but also not sudden, would have instigated anxiety and insecurity among at least segments of various communities. Such perception was limited to their own spatial stories, perhaps complemented by secondhand stories from other commoners that could have been encountered sporadically at markets.

In contrast, as noted above, elites beginning in the Epi-Classic period at the latest had more regional and extra-regional contacts by which they acquired foreign objects. Furthermore, the documentary record indicates that as part of their political and religious practices, elites were occupying higher elevations. At least retrospectively in a mytho-historical narrative of the founding of the Tarascan State (de Alcalá 2000), hereditary elites in the late pre-Hispanic period regularly traveled to elevated spaces, particularly mountaintops. The three most prominent reasons for this are bellicose political actions (making bonfires and “sending a message” to those who could view them and marking territory), religious experience (encountering the gods), and gathering firewood (in addition to its mundane uses discussed above, wood was necessary to keep temple fires going as a means of pleasing and/or communicating with the gods). The name the elite in the Tarascan State referred to themselves with is also explicitly linked to higher elevations—the royal lineages were collectively known as *Uacúsecha*, meaning “eagles.”

Such actions by the elites would have also led to their ability to gain an elevated vantage point from which to view the changing landscape. More than mapping the spatiotemporal events in relation to specific places of the basin, elites would have been able to perceive the landscape more as we perceive a map, as a unifying vision of the places and spaces of the basin. By incorporating the eventfulness of the changes as marked by the places of the basin’s landscape, such elites would have been able to integrate these spatial and temporal understandings of the landscape to envision the totality of consequences of continued changes at various rates within memory.

Such practices did not exist in a social vacuum, however. We suggest it was the relation between these time-space maps formulated by the elites and the spatial stories of the commoners that was key to mobilizing community action, action which of course solidified the place of the elites and gave them new opportunities to bring other communities under their sway. As stated above, the spatial stories and the changes in the landscape affecting these stories would have led to resource anxiety or at the very least changes at certain points in time of the very nature of those spatial stories, e.g., a transition from land travel to canoe travel. Furthermore, these perceptions and community mobilization would have been all the more pressing in the Early, Middle, and Late Postclassic periods as lake regressions and transgressions first revealed or made possible and then submerged not merely landscape features or resource zones but communities or settlements themselves (for example the aforementioned dwellings on those *lomas* surrounding Jarácuaro). Elites would have been in a prime position, due to their more totalizing perceptions of space and its temporalities, to formulate a framework by which the commoners could understand the nature or the full import of their own limited perceptions. In this way, we suggest that the mutual but asymmetrical extent to which the landscape was perceived by elites and commoners would have authenticated and reinforced one another. Within this relationship, we can posit yet another asymmetry of power that was constituted not by the mere difference of perception and cognition but further by the ability, grounded in their superior perception, to integrate, orient, and organize the disparate and incomplete perceptions of the commoners. Within this relationship that was asymmetrical but in which the commoners played an important role, communal action would have been easier to mobilize and cast as a necessary course in the possible futures being formulated and represented by the elites. Elites became not simply managers of people but managers of people, of places, of things (namely resources), of time and futures, and of the mutual but shifting nature of the constitution of each of these by the others (following Dillehay 2014, pp. 296–297).

In this way, elites solidified their elite status *vis-à-vis* commoners in their communities but also mobilized the members of their communities for different courses of action. One of those courses of action was of course the organized violence by which elites competed with other elites. In addition, and more in line with the elites’ role as managers of time and temporalities manifested in economic and social behaviors as well as the changing landscape, the role of communal projects to increase agricultural productivity in the form of terracing projects documented by Fisher (2005; also Pezzutti 2010) needs to be addressed by future research. These terraces require more research and particularly more secure dating, but it is essential to consider them and their construction and subsequent maintenance as mitigations against some futures and attempts to bring others to fruition. Fisher’s (2005) interest is in the post-conquest resiliency (or lack thereof) of the landscape of the LPB, and in particular its agrarian system with these terraces, as impacted by the loss of innumerable indigenous persons during the early years of Spanish colonialism. We in turn point out that such terraces are likely telling just in terms of their existence, signifying their construction at some point in the past. Their location, associated loosely with Late Postclassic settlements, indicates that they were part of the practices whereby communities, their governmental apparatuses, and a growing supra-community level state apparatus sought to provide for a future in which arable land was or would become scarce (which obviously did happen). The fact that slope land was invested in is grounded in a recognition that lake and basin floor economic and subsistence adaptations would become less feasible over time. They are materializations of the perception of the need or at least desirability of such terraces as well as the time management of the labor that would have been required to construct and maintain them. In other words, these terraces as well as other potential shifts in subsistence and economy are not notable merely as adaptations but as reflections of allocations of labor in time and over time, perceptions of temporal changes, and mitigations against potential negative futures and movements toward more positive futures (following Sassaman 2012). Particularly with population growing (Pollard 2008; see above), they were, as much as present evidence suggests, part of a strategy of multiple solutions to sustaining the population of the LPB that was embarked upon.

Integrating these various observations and data indicates or is strongly suggestive that elites’ role in “managing time” extended beyond merely perceiving and representing a changing landscape and its implications and beyond merely organizing that information to incite inter-community violence but also included managing the time and labor of the community in subsistence and economic activities that would have augmented the community’s future sustainability in multiple possible futures. Whether these labor commitments manifested in terraces occurred before, simultaneously with, or after political consolidation of the LPB and the wider region is, again, an important point for future research to resolve. A “warrior” ideology and mentality among the elite that dated to at least the Early Postclassic in the LPB (Pollard and Cahue 1999) was likely easily augmented as these elites were able to mobilize their communities and transform the nature and consequences, or at the very least the outcomes, of organized violence such that conquest, tribute exaction, and labor obligations became the result.

## Landscape, Futurity, and Social Transformations in Macroregional Perspective

The theoretical argument posed when discussing the political and social transformations that gave rise to the Tarascan State in this study can be extrapolated to a macroregional scale by including a similar discussion of the data pertaining to the Zacapu Basin, which lies approximately 20 km to the northwest of the LPB. In this regard, we do not have specific data but rely on the publications of the Zacapu Project carried out by the *Centre d’Etudes Mexicaines et Centro-Americaines* (CEMCA). Reconstructions of the settlement systems show that during the Pre-Classic, Zacapu Basin communities “appeared to have been primarily adapted to lacustrine ecosystems, locating their villages either on island marshes or along lakeshore or rivers” (Pollard 1997, p. 360). In other words, initial settlement of the Zacapu Basin displays similar subsistence and community patterns to that of the early phases of the LPB. However, this is the last instance when the Zacapu communities relied primarily on these resource zones and this settlement strategy. The Classic period marks drastic change for the communities of this basin, as they begin to move away from the lakeshore and form communities in the slope zone. Also during the Classic period, settlements expand and multiply as population increases (Michelet 2008). This trend continues into the Terminal Classic, with population continuing to increase, and settlement spreading throughout the slope zones of the Zacapu Basin, away from marsh and lake resources (Pollard 1997). The Postclassic continues the settlement of the slopes of Zacapu and introduced a new occupation, although still relatively small, in the malpaís of Zacapu. It is during the Early Postclassic and into the Middle Postclassic that communities nucleated, inhabiting defensible locations. Populations and settlement begin to grow at a rapid rate in the malpaís during the Middle Postclassic, as aggressive populations continued to move in and out of the basin, competing for resources (Michelet 2008, p. 597). Sites are abandoned and re-settled, displaying a level of unrest and settlement disruption during this time.

The emergence of the state and beginning of the Late Postclassic saw the populations remain in the malpaís, and grow to very large numbers, in both community size and population. This increase in population and continued nucleation of settlements suggest two things. The first is stability and the lack of warfare with the emergence of the state, allowing for more permanent and large-scale settlements. Second, the emergence of the state introduced a new political economy, from which the large settlement of El Palacio benefitted, as the upper-level elites aligned themselves with the Tarascan elites of the LPB, and participated in long-distance exchange, state ritual and further developed the complex hierarchical social system that was most likely in place since the Classic period.

Once again, community mobilization and elite strategy for settlement play a large role in how and where the Zacapu populations move to/from. In this case, warfare and an unstable political system seem to play a large role in the Zacapu Basin and threatened the power and control of the local elites. The move away from the lacustrine zones early on in the temporal sequence displays a seemingly drastic change, one that disrupts the accumulated memory of and relationship with the then current perceived landscape—a direct contrast with the data for the LPB in which communities were much more stable and remained oriented toward the lake and its resources. And while this seemingly radical change does emphasize a large disadvantage for community, it also highlights the necessity for community survival by moving to defensible, higher elevated settlements. Agency and decision-making at the community level can be seen in this instance, and as researchers, we can observe this as a protended action, based on past or present circumstances and memories for future paths.

Similar to the “generational” mapping of landscape perception in the LPB, we must adopt a similar view of the Zacapu Basin. While, in archaeological terms, these settlement changes of the Zacapu Basin seem drastic and sudden, this is far from the case. As discussed by Michelet (2008, p. 597), it was not until the Middle Postclassic that the lake marsh zone was almost completely uninhabited, which means that the move away from the lakeshore took approximately 300 years. And even with the change in settlement to more elevated locations, such as the farther north slope zone that ultimately borders the Lerma River Valley or the malpaís of Zacapu, researchers note that within these areas the settlement patterns fluctuate, as they are abandoned and re-occupied throughout the Middle Postclassic into the Late Postclassic (Arnauld and Faugère-Kalfon 1998; Migeon 2003). In essence, the social and political instability and the ebb and flow of warfare throughout the region act as a catalyst for change, similar to the fluctuations of Lake Pátzcuaro. As settlement changes and communities shift, they act upon the informed memory of the landscape in order to continually adapt to the present situations, using their past knowledge to dictate decision making for the future.

Finally, there is the issue of even larger scale demographic change, as the northern “frontier” of Mesoamerica shifted northward during the Epi-Classic and Early Postclassic only to regress back to its more normal latitude in the Middle and Late Postclassic. Such shifts, particularly the latter regression, encouraged demographic shifts southward as farming was rendered problematic in the expanded frontier zone. While the ethnic affiliations of the migrating peoples, their numbers, and the specific regions that various groups migrated into are debated, the archaeological record concerning sites in the expanded Epi-Classic/Early Postclassic frontier zone and their abandonment in the Middle and Late Postclassic is clear. We cannot say for certain whether these groups migrated into the Pátzcuaro Basin, for example (or whether demographic expansion in the basin could have been internal, i.e., due to birth rates outpacing death rates), but such movements and the potential impact of movements of people into the Lake Pátzcuaro Basin could have also impacted decision making at the local scale and the garnering of support for resource motivated wars.

## Conclusion

GIS technology has enabled us to create and utilize new ways of envisioning the dynamism of the Lake Pátzcuaro Basin landscape in the past. These representations, viewed through the lens of phenomenological philosophy, allow us to see time in the landscape as marked by its various features. This approach and the influence of “time consciousness” move us past the equally problematic if antithetical practices of “inhabiting” the past either through the lens dominated by a particular instant in the past or the lens of archaeologically produced time maps in which time as represented by archaeological materials is chopped into discontinuous units of time which are sometimes problematically related to one another in terms of their transitions. However, human experience of temporality is not a ceaseless flow in which the past is inevitably gone and the future is unknowable but rather always a process of bringing the past into the present and envisioning the future. The futurity of perception and practice has not been emphasized in many applications of phenomenology, nor of GIS, in archaeology. Here we have taken initial steps to rectify that situation. Furthermore, by indicating that investigating social processes necessarily involves envisioning social actors as historical beings, dwelling in the landscape through time, and that this practice of dwelling partially constituted their agency and dispositions toward any particular course of action, we have charted one way to investigate human eco-dynamics without reverting to an ecological approach that separates and reifies the environment (which GIS applications have been critiqued for supporting) and that avoids ignoring the landscape altogether in a form of extreme “social determinism” (Algaze 2001).

As discussed by Llobera (2012), the perceived theoretical and methodological gap between GIS applications and interpretive archaeologies of landscape, such as a phenomenological one undertaken here, are likely overblown. Of course direct perception of the landscape is preferable, but direct perception of past landscapes is by definition impossible. We can gain entry to those past landscapes through archaeological fieldwork, analysis of artifactual remains and landscape features, through narratives, and as we have shown through GIS applications and representations. Directly perceiving the landscape *with* other subjects, who possess and share narratives about the landscape with others, reveals the simultaneous possibility and impossibility of perceiving past landscapes in the present. We have not been able to directly perceive Jarácuaro as an island as we have stood at various vantage points in the LPB, but by listening to others who are older and wiser and can remember and “re-present” those memories for us, we can imagine the landscape in our minds and the myriad consequences of the lake extending to this landmark but not that landmark.

The narrative with which we opened is not merely anecdotal; we posit some homology between our direct experiences and our “received experiences” through such representations of elder mentors with the experiential and active dwelling of the past inhabitants of this same landscape that we seek to understand. Note that such “re-presentation” in the presence and active perception of the landscape which itself structures the content of those representations is different from the kind of passive and detached representation mediated solely by technological means that Thomas (2004) and Tilley (1994, 2004) decry. Furthermore, and grounded in excellent ethnographic and ethnoarchaeological work in the LPB regarding various communities of practice and the exploitation of numerous ecological niches that the landscape provides (Williams 2014), we believe it is abundantly clear that actual inhabitants of the basin, past and present, would have been far better at perceiving and experiencing the landscape of the LPB than we are, having moved over its terrain in daily, seasonal, yearly, etc. rounds. We can only inhabit representations of past landscapes (as in Fig. 3a–f) but with enough of the other kinds of knowledge regarding ecological and economic behaviors of contemporary inhabitants and archaeological reconstructions of past inhabitants (e.g., Hirshman and Stawski 2013), it is easier to posit how landscape changes would have affected past inhabitants of the LPB. Such experiences, remembrances, and representations attune our eyes and cognitive capacities to what kinds of changes and what places might garner our attention, what places are or might become salient to our lives given past, present, and future changes. In this way, direct experience of the landscape, familiarity with its topography and ecology, historical or temporal recollections and narratives, ethnography, and ethnoarchaeology all combine to enhance our appreciation of the necessity of perceiving not simply place but time, temporality, and even directionality in the landscape. Those sources of information then aid us in the perception and virtual inhabitation of past landscapes using GIS, methods of landscape reconstruction aided by archaeological data, and viewshed representations of past landscapes informed by phenomenological philosophy.

In so doing, we can see how past inhabitants would have appreciated not only temporalities as they relate to place (and place as it is manifested temporally) in the present and the past; additionally and along with Sassaman (2012), we posit that any perception of present and past necessarily implicated potential futures that variously instigated future-oriented action. It was future-oriented action, action built out of the mutual interpenetration of the landscape and perceiving and knowledgeable inhabitants thereof that undertook the range of practices by which the Tarascan State took shape.

Finally, while archaeologists should at times be wary of representations as only pale or inadequate stand-ins for the “real thing,” a prominent strain within phenomenological thought as it has been applied in archaeology, we should not be so dismissive of representations out of hand. Representations are at least ways that we in the present can inhabit past landscapes and do so temporally, as we have demonstrated here. They are not as good as being there, perhaps, but in many analytic contexts, they are the best we have. To the same extent, it is possible that representation and experience (“being” or “inhabiting”) are not as antithetical as has been supposed. Such processes can in many instances instigate cognition and the production of abstract representations, which to keep with the theme of this paper would be subsumed with the imaginary inhabitation of potential worlds that is nonetheless based on direct perception of the present and in coordination with the incorporation of the past via memory, narrative, and other representations. This relationship between subjective perception and more totalizing representations is after all precisely what Gell (1992) discusses in his reconciliation of A-series and B-series time. It is possible that the emphasis of direct experience within phenomenology that a few archaeologists at least have emphasized while eschewing modern technology (e.g., Tilley 1994, 2004; Thomas 2004; see discussion in Llobera 2012) is overwrought and runs the risk of only emphasizing the “now” and the “direct” at the expense of the “possible,” the “imaginary,” and the “merely representational.” Again, such an approach is at odds with a process of inhabitation that is also informed by modes of active reflection that we suggest were spurred by the landscapes themselves especially as they were engaged in human projects of inhabitation.

## Electronic Supplementary Material

Below is the link to the electronic supplementary material.

ESM 1 (MP4 9502 kb)

## Footnotes

1 We by no means wish to implicate Helen in our own reflective shortcomings. Her work, due in large part to her training but likely also aided by her having witnessed large-scale fluctuations of the lake (as opposed to our limited experience over our young careers), has always been temporally oriented by including change and adaptation within it. It is only due to lack of evidence, which she is to be recognized for rectifying, that in her early publications the lake is depicted repetitively as it existed at the time of Spanish contact.

2 The one factor not accounted for in the landscape reconstruction is the impact and alteration of the landscape from centuries of soil erosion and soil redeposition. This especially affects lower lying areas adjacent to stream/riverine systems and the lake. As Fisher et al. (1999, 2003) and Fisher (2005) have documented, however, there were only minor and very localized erosion events in the pre-Hispanic sequence, mainly during the Late Classic and Epi-Classic periods. There has been a debate between Fisher and some limnologists over whether major and widespread erosional deposition occurred before or after the Spanish conquest and was caused by either the non-sustainable practices during the Late Postclassic brought on by population pressure or decline in population after the conquest due largely to infectious diseases, which led to the inability to keep up investments in the landscape, namely terraces.

3 Unfortunately, Heidegger is not much help in analyzing such transformations between readiness-to-hand and present-at-hand, or other possible relations between these engagements, over time. Harman’s (2002, p. 65) discussion and critique note that “time” is never really the focus of Heidegger’s philosophical probing at all. Perhaps the philosophical formulation of a culturally adapted readiness to hand that incorporates change and thus present-at-handness spurred by reflection as a result of such change can be formulated; our focus lies elsewhere.

4 It is difficult to exactly quantify such changes because it is largely unknown at this point where potters were producing their wares, e.g., if there were significant numbers of potters in Jaracuaro and its environs.

## References

[CR1] Algaze G (2001). Initial social complexity in Southwest Asia. Current Anthropology.

[CR2] Arnauld, M., & Faugère-Kalfon, B. (1998). Evolución de la ocupación humana en el Centro-Norte de Michoacán (Proyecto Michoacán, CEMCA) y la emergencia del Estado Tarasco. In *Génesis, culturas y espacios en Michoacán*, V. Darras (Ed.), pp. 13-34. Centre d’Etudes Mexicaines et centraméricaines, Mexico.

[CR3] Arnold PJ, Holdaway S, Vandsnider LA (2008). No time like the present. Time in archaeology: time perspectivism revisited.

[CR4] Ashmore, W., & Knapp, A. B. (Eds.). (1999). *Archaeologies of landscape: contemporary perspectives*. New York: Wiley.

[CR5] Basso K (1996). Wisdom sits in places: landscape and language among the Western Apache.

[CR6] Bender B (1993). Landscape: perspective and politics.

[CR7] Bradbury JP (2000). Limnologic history of Lago de Pátzcuaro, Michoacán, Mexico for the past 48,000 years: impacts of climate and man. Palaeogeography Palaeoclimatology Palaeoecology.

[CR8] Brivio PA, Pepe M, Tomasoni R (2000). Multispectral and multiscale remote sensing data for archaeological prospecting in an alpine alluvial plain. Journal of Cultural Heritage.

[CR9] Brumfiel, E. M. (1992). Distinguished lecture in archaeology: breaking and entering the ecosystem--gender, class, and faction steal the show. *American Anthropologist**94*(3), 551–567.

[CR10] Casey E, Feld S, Basso KH (1996). How to get from space to place in a fairly short stretch of time: phenomenological prolegomena. Senses of place.

[CR11] Connerton P (1989). How societies remember.

[CR12] Cuelenaere L (2011). Aymara forms of walking: a linguistic anthropological reflection on the relation between language and motion. Language Sciences.

[CR13] de Alcalá, F J. (2000). *Relación de Michoacán*. Moisés Franco Mendoza, coord., with paleography by Clotilde Martínez Ibáñez and Carmen Molina Ruiz. Zamora, Mexico: El Colegio de Michoacán and Gobierno del Estado de Michoacán.

[CR14] De Certeau M (1984). The practice of everyday life.

[CR15] Dillehay TD (2014). The teleoscopic polity: Andean patriarchy and materiality.

[CR16] Fisher, C. (2000). Landscapes of the Lake Pátzcuaro Basin. PhD dissertation, University of Wisconsin-Madison.

[CR17] Fisher C (2005). Demographic and landscape change in the Lake Patzcuaro Basin, Mexico: abandoning the garden. American Anthropologist.

[CR18] Fisher C, Pollard H, Frederick C (1999). Intensive agriculture and socio-political development in the Lake Pátzcuaro Basin, Michoacán, Mexico. Antiquity.

[CR19] Fisher C, Pollard HP, Israde-Alcántara I, Garduño-Monroy VH, Banerjee SK (2003). A reexamination of human-induced environmental change within the Lake Pátzcuaro Basin, Michoacán, Mexico. Proceedings of the National Academy of Sciences of the United States of America.

[CR20] Foucault M (1971). The order of things.

[CR21] Gell A (1992). Anthropology of time: cultural constructions of temporal maps and images.

[CR22] Gomez-Tagle Chavez A, Bernal-Brooks FW, Alcocer J (2002). Sensitivity of Mexican water bodies to regional climatic change: three study alternatives applied to remote sensed data of Lake Pátzcuaro. Hydrobiologia.

[CR23] González RM (2010). La Dimensión Mítica de la Peregrinación Tarasca. Journal de la Sociètè des Amèricanistes.

[CR24] Gorenstein, S., & Pollard, H. (1983). The Tarascan civilization: a late Prehispanic cultural system. Vanderbilt University Publications in Anthropology, No. 28, Nashville, TN.

[CR25] Hägerstrand, T. (1973). The domain of human geography. In: *New directions in geography.* Chorley, R. (Ed.), pp. 67–87. Cambridge: Cambridge University Press.

[CR26] Harman G (2002). Tool-Being: Heidegger and the ontology of objects.

[CR27] Harris M (1998). The rhythm of life on the Amazon floodplain: seasonality and sociality in a riverine village. Journal of the Royal Anthropological Institute (N.S.).

[CR28] Harris M (2005). Riding a wave: embodied skills and colonial history on the Amazon floodplain. Ethnos.

[CR29] Harvey D (1990). The condition of postmodernity: an enquiry into the origins of cultural change.

[CR30] Haskell DL (2013). Tarascan historicity: narrative structure and the production of the past in the case of the two Tariacuris. Ethnohistory.

[CR31] Heidegger, M. (1962). *Being and time*. Translated by John Macquarrie & Edward Robinson. San Francisco: Harper.

[CR32] Hirsch E (2006). Landscape, myth and time. Journal of Material Culture.

[CR33] Hirshman AJ, Ferguson JR (2012). Temper mixture models and assessing ceramic complexity in the emerging Tarascan State. Journal of Archaeological Science.

[CR34] Hirshman A, Stawski C (2013). Distribution, transportation, and the persistence of household ceramic production in the Tarascan State. Ethnoarchaeology.

[CR35] Hirshman AJ, Lovis WA, Pollard HP (2010). Specialization of ceramic production: a sherd assemblage based analytic perspective. Journal of Anthropological Archaeology.

[CR36] Hritz C (2010). Tracing settlement patterns and channel systems in southern Mesopotamia using remote sensing. Journal of Field Archaeology.

[CR37] Ingold T (1993). The temporality of the landscape. World Archaeology.

[CR38] Ingold T (2004). Culture on the ground: the world perceived through the feet. Journal of Material Culture.

[CR39] Israde-Alcántara I, Garduño-Monroy VH, Fisher CT, Pollard HP, Rodríguez Pascua MA (2005). Lake level change, climate and the impact of natural events: the role of seismic and volcanic events in the formation of the Lake Pátzcuaro Basin, Michoacán, Mexico. Quaternary International.

[CR40] Jones ET (2006). Using viewshed analysis to explore settlement choice: a case study of the Onondaga Iroquois. American Antiquity.

[CR41] Koselleck, R. (2004). *Futures past: on the semantics of historical time*. New York: Columbia University Press.

[CR42] Lave J, Wenger E (1991). Situated learning: legitimate peripheral participation.

[CR43] Leone M, Shackel P, Ingersoll D, Bronitsky G (1987). Forks, clocks and power. Mirror and metaphor: material and social construction of reality.

[CR44] Llobera M (2003). Extending GIS-based visual analysis: the concept of visualscapes. International Journal of Geographic Information Science.

[CR45] Llobera M (2012). Life on a pixel: challenges in the development of digital methods within an “interpretive” landscape archaeology framework. Journal Archaeological Method and Theory..

[CR46] Lucas G (2005). Archaeology of time.

[CR47] Merleau-Ponty M (1962). Phenomenology of perception.

[CR48] Metcalfe S, Davies S (2007). Deciphering recent climate change in central Mexican lake records. Climatic Change.

[CR49] Metcalfe S, Davies SJ, Braisby JD, Leng MJ, Newton AJ, Terrett NL, O’Hara SL (2007). Long and short-term change in the Pátzcuaro Basin, central Mexico. Palaeogeography Palaeoclimatology Palaeoecology.

[CR50] Michelet, D. (2008). Living differently. The sites of the Milpillas phase (1250–1450 A.D.) in the Malpaís de Zacapu (Michoacán). In: *Urbanism in Mesoamerica*, vol. 2, A.G. Mastache, R. Cobean, A. Cook, K. Hirth (Eds.), pp. 593–620. Penn State Publications, INAH Publications.

[CR51] Migeon G (2003). Abandonos planificados, rituales de vasijas matados o de clausura y ocupaciones posteriores: Los sitios del cerro Barajas, Guanajuato y de Milpillas, en el Malpais de Zacapu, Michoacán. Trace.

[CR52] Morphy, H. (1995). Landscape and the reproduction of the ancestral past. In E. Hirsch & M. O’Hanlon (Eds.), *The anthropology of landscape: perspectives on place and space,* pp. 184–209. Oxford: Clarendon.

[CR53] Munn N (1996). Excluded spaces: the figure in the Australian aboriginal landscape. Critical Inquiry.

[CR54] O’Hara, S. (1993). Historical evidence of fluctuations in the level of Lake Pátzcuaro, Michoacán, Mexico over the last 600 years. *The Geographic Journal*, *159*(1), 51–62.

[CR55] O'Hara, S. L., Street-Perrott, F. A., & Burt, T. P. (1993). Accelarated soil erosion around a Mexican highland lake cause by prehispanic agriculture. *Nature*, 363, 48–51.

[CR56] Olsen B (2010). In defense of things: archaeology and the ontology of objects.

[CR57] Pezzutti, F. (2010). *The steps of kings: terraced landscapes in the Lake Patzcuaro Basin, Michoacan, Mexico*. Unpublished MA Thesis, Colorado State University, Department of Anthropology.

[CR58] Pollard H (1980). Central places and cities: a consideration of the protohistoric Tarascan State. American Antiquity.

[CR59] Pollard H (1982). Ecological variation and economic exchange in the Tarascan State. American Ethnologist.

[CR60] Pollard H (1997). Recent research in west Mexican archaeology. Journal of Archaeological Research.

[CR61] Pollard, H. (2000). Proyecto Desarollo del Estado Tarasco: Los Senorios Urichu, Xaracuaro, y Pareo. Informe Final al Consejo de Arqueologia, Instituto Nacional de Antropologia e Historia.

[CR62] Pollard H (2008). A model of the emergence of the Tarascan State. Ancient Mesoamerica.

[CR63] Pollard H, Roth-Seneff A, Kemper RV, Adkins J (2015). The Prehispanic heritage of the Tarascans (Purépecha). From tribute to communal sovereignty: the Tarascan and Caxcan territories in transition.

[CR64] Pollard HP, Cahue L (1999). Mortuary patterns of regional elites in the Lake Pátzcuaro Basin of western Mexico. Latin American Antiquity.

[CR65] Pred A (1977). The choreography of existence: comments on Hägerstrand’s time-geography and its usefulness. Economic Geography.

[CR66] Sassaman KE (2012). Futurologists look back. Archaeologies: Journal of the World Archaeological Congress.

[CR67] Stawski, C. (2012). Settlement systems, landscapes and the rise of the Tarascan empire: a settlement analysis in the Lake Pátzcuaro Basin, Michoacán, Mexico. PhD Dissertation, Michigan State University, East Lansing, MI.

[CR68] Thomas J (2004). Archaeology and modernity.

[CR69] Thompson EP (1967). Time, work-discipline, and industrial capitalism. Past and Present.

[CR70] Tilley C (1994). A phenomenology of landscape.

[CR71] Tilley C (2004). The materiality of stone: explorations in landscape phenomenology.

[CR72] Van der Leeuw S, Longacre WA (1991). Variation, variability, and explanation in pottery studies. Ceramic ethnoarchaeology.

[CR73] Van Leusen M (1999). Viewshed and cost surface analysis using GIS (cartographic modelling in a cell-based GIS II). BAR International Series.

[CR74] Watts WA, Bradbury JP (1982). Paleoecological studies at Lake Pátzcuaro on the west-central Mexican plateau and at Chalco in the basin of Mexico. Quaternary Research.

[CR75] West, Robert C. (1948). *Cultural geography of the modern Tarascan area*. Institute of Social Anthropology Publication 7. Washington, DC: Smithsonian Institution

[CR76] Whitington J (2013). Fingerprint, bellwether, model event: climate change as speculative anthropology. Anthropological Theory.

[CR77] Williams, E. (2014). Water folk: reconstructing an ancient aquatic lifeway in Michoacán, Western Mexico. British Archaeological Reports, International Series, 2617, Oxford, UK.

[CR78] Zedeño MN, Ballenger JAM, Murray JR (2014). Landscape engineering and organizational complexity among late prehistoric bison hunters of the Northwestern Plains. Current Anthropology.

